# Antimicrobial activity of apple cider vinegar against *Escherichia coli*, *Staphylococcus aureus* and *Candida albicans*; downregulating cytokine and microbial protein expression

**DOI:** 10.1038/s41598-017-18618-x

**Published:** 2018-01-29

**Authors:** Darshna Yagnik, Vlad Serafin, Ajit J. Shah

**Affiliations:** Department of Natural Sciences, School of Science and Technology, Middlesex University, The Burroughs, London, NW4 4BT England United Kingdom

## Abstract

The global escalation in antibiotic resistance cases means alternative antimicrobials are essential. The aim of this study was to investigate the antimicrobial capacity of apple cider vinegar (ACV) against *E. coli, S. aureus* and *C. albicans*. The minimum dilution of ACV required for growth inhibition varied for each microbial species. For *C. albicans*, a 1/2 ACV had the strongest effect, *S. aureus, a* 1/25 dilution ACV was required, whereas for *E-coli* cultures, a 1/50 ACV dilution was required (p < 0.05). Monocyte co-culture with microbes alongside ACV resulted in dose dependent downregulation of inflammatory cytokines (TNFα, IL-6). Results are expressed as percentage decreases in cytokine secretion comparing ACV treated with non-ACV treated monocytes cultured with *E-coli* (TNFα, 99.2%; IL-6, 98%), *S. aureus* (TNFα, 90%; IL-6, 83%) and *C. albicans* (TNFα, 83.3%; IL-6, 90.1%) respectively. Proteomic analyses of microbes demonstrated that ACV impaired cell integrity, organelles and protein expression. ACV treatment resulted in an absence in expression of DNA starvation protein, citrate synthase, isocitrate and malate dehydrogenases in *E-coli*; chaperone protein DNak and ftsz in *S. aureus* and pyruvate kinase, 6-phosphogluconate dehydrogenase, fructose bisphosphate were among the enzymes absent in *C.albican cultures*. The results demonstrate ACV has multiple antimicrobial potential with clinical therapeutic implications.

## Introduction

Antibiotic resistance is rapidly becoming a major worldwide problem. There has been a steady increase in the number of pathogens that show multiple drug resistance. In fact the World Health Organization predicts that infections involving antibiotic resistant pathogens will pose major patient care management issues in the future^1^.This will inevitably lead to an increase in hospital stays, cost, patient morbidity and mortality. In the immunocompromised and at risk patients severe microbial infections can result in sepsis. Sepsis can rapidly lead to systemic inflammation and organ failure^2^. In response to microbial invasion, the innate immune system reacts by triggering tissue damage. Mononuclear cells recognize pathogens associated with molecular patterns (PAMPs) present on the microbial surface. This results in intracellular signaling cascades which initiate pro-inflammatory cytokine and chemokine release into the blood circulation. Unchecked, the chemokines will continue to recruit more immune cells to the site of infection which release further pro-inflammatory cytokines enhancing inflammation in a continuous feedback loop^3^. Essentially, antibiotics are antimicrobials but can also act as immune modulators reducing the release of pro-inflammatory cytokines such as IL-1β, IL-6, TNFα, IL-8, and interferon-gamma (INF-gamma). Antibiotics can also affect mononuclear phagocytic function and modulate the activity of nuclear transcription factors such as NF-ĸB and activator proteins^4–7^. Microorganisms such as *E-coli, S. aureus* and *C. albicans* form part of the human microbiota. However pathogenic forms of these microbes have been implicated in blood or urinary tract infections, gastroenterititis, endocarditis, soft tissue infections and organ malfunction^8–10^.

The anti-microbial agents used to treat gram negative infections such as β-lactams, fluroquinolones, sulfamethaoxathoxazole and trimethroprin are becoming increasingly ineffective. Strains of *S. aureus* have emerged with reduced susceptibility to vancomycin and methicillin^6,11,12^. Furthermore, antibiotic action itself can be problematic in terms of cell membrane permeability, intracellular inactivation and the inability to reach intracellular structures in which organisms can hide. Alternative supplementation that can combat a plethora of microbes without concurrent side effects would be of significant healthcare interest as the discovery of effective new antibiotic has been slow but should be a global priority.

The Old Testament and Hippocrates reported on the use of ACV in combination with honey to combat infection and protect open skin wounds. Historically, vinegar has been produced and sold as a commercial commodity for over 5000 years. In fact up until the sixth century BC, the Babylonians were making vinegars for consumption as well as for use in healing^13^. Vinegar is the resultant product when ethyl alcohol is converted to acetic acid by Acetobacter. It can be produced by different methods and a variety of raw materials such as wine, malted barley, alcohol, fruits and cider^14^. ACV is produced from cider that has undergone acetous bioconversion and has relatively low acidity (5% acetic acid). It also contains organic acids, flavonoids, polyphenols, vitamins and minerals^15^. ACV has been hailed as a supplement aiding weight loss, hyperlipademia, hypercholesterliaemia, nutritional support, antioxidant defence and lowering blood pressure. Utilising organic acids as nutritional supplements has been regarded as safe and can eliminate harmful intestinal bacteria^16–18^. The positive impact of dietary ACV supplementation has been highlighted *in vivo*. ACV decreased the serum lipid profile in mice fed a high cholesterol diet over 28 days. Intragrastric ACV addition induced a protective effect against erythrocyte, kidney and liver oxidative injury as well as lowering cholesterol levels^16^. ACV also decreased blood triglyceride and very low density lipoprotein levels in rats which had induced cholesterol induced hepatic steatosis^18^. Despite the known health benefits of dietary organic acid supplementation, to the best of our knowledge the direct effect of ACV on microbes and mononuclear leucocytes has not been examined. The aim of the present study was to investigate the antimicrobial activity of ACV on microbes and associated inflammatory pathways.

## Results

### The antibacterial and antifungal activity of ACV against *E*. *coli*, *S*. *aureus* and *C*. *albicans*

**Figure 1:**
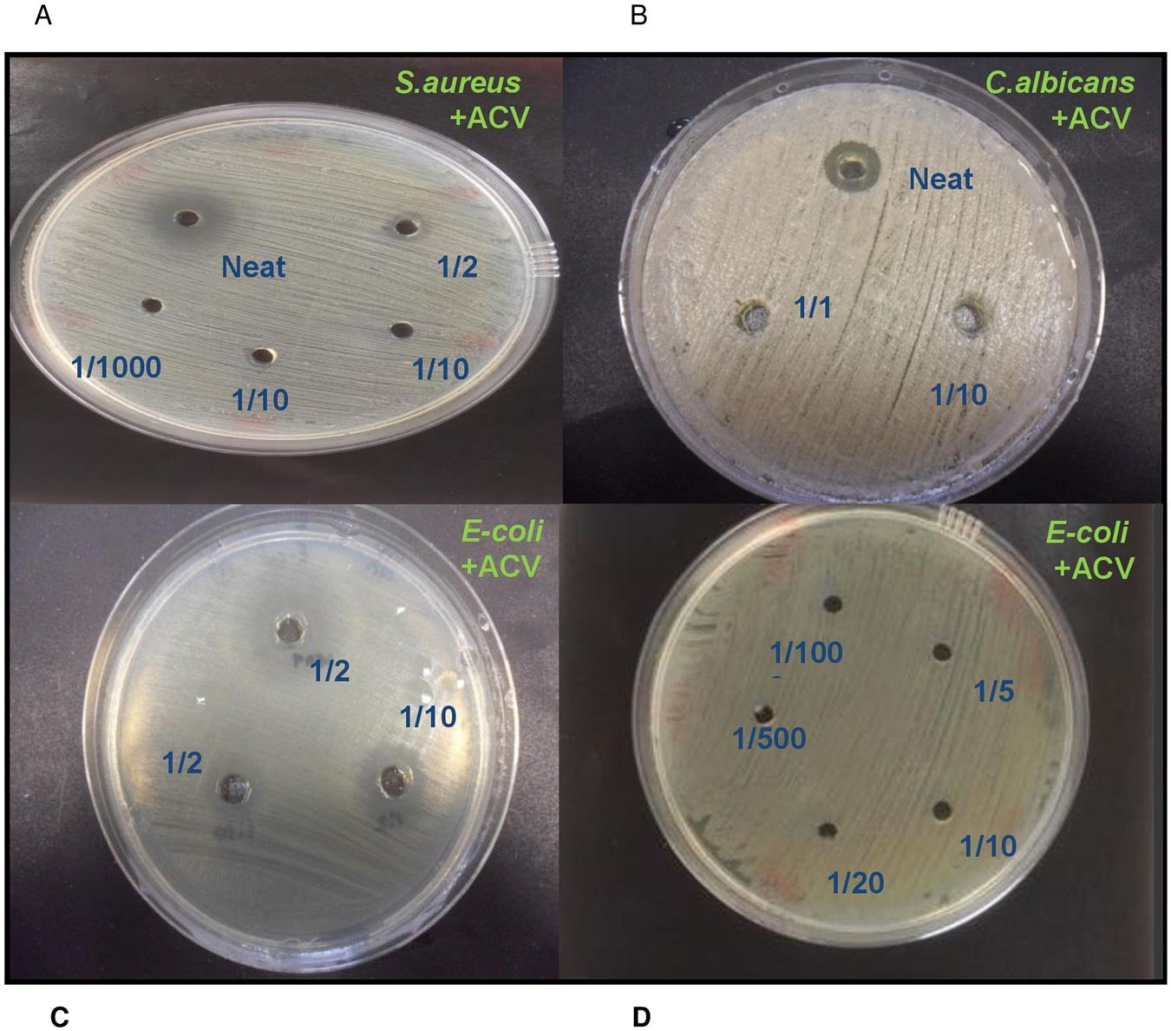
Effect of varying concentrations of ACV on microbial growth after incubation at 37 °C for 24 h. (**a**) *S. aureus*; (**b**) *C*. *albicans*, (**c**) *E-coli* (**d**) *E-coli*. ACV was either applied neat or diluted 1:2 or 1:10 v/v in distilled water. Zones of microbial growth inhibition are indicated by clear zones and vary with ACV dilutions for each microbe. Photographs were taken using a 20 Mega pixel Samsung camera.

**Figure 2:**
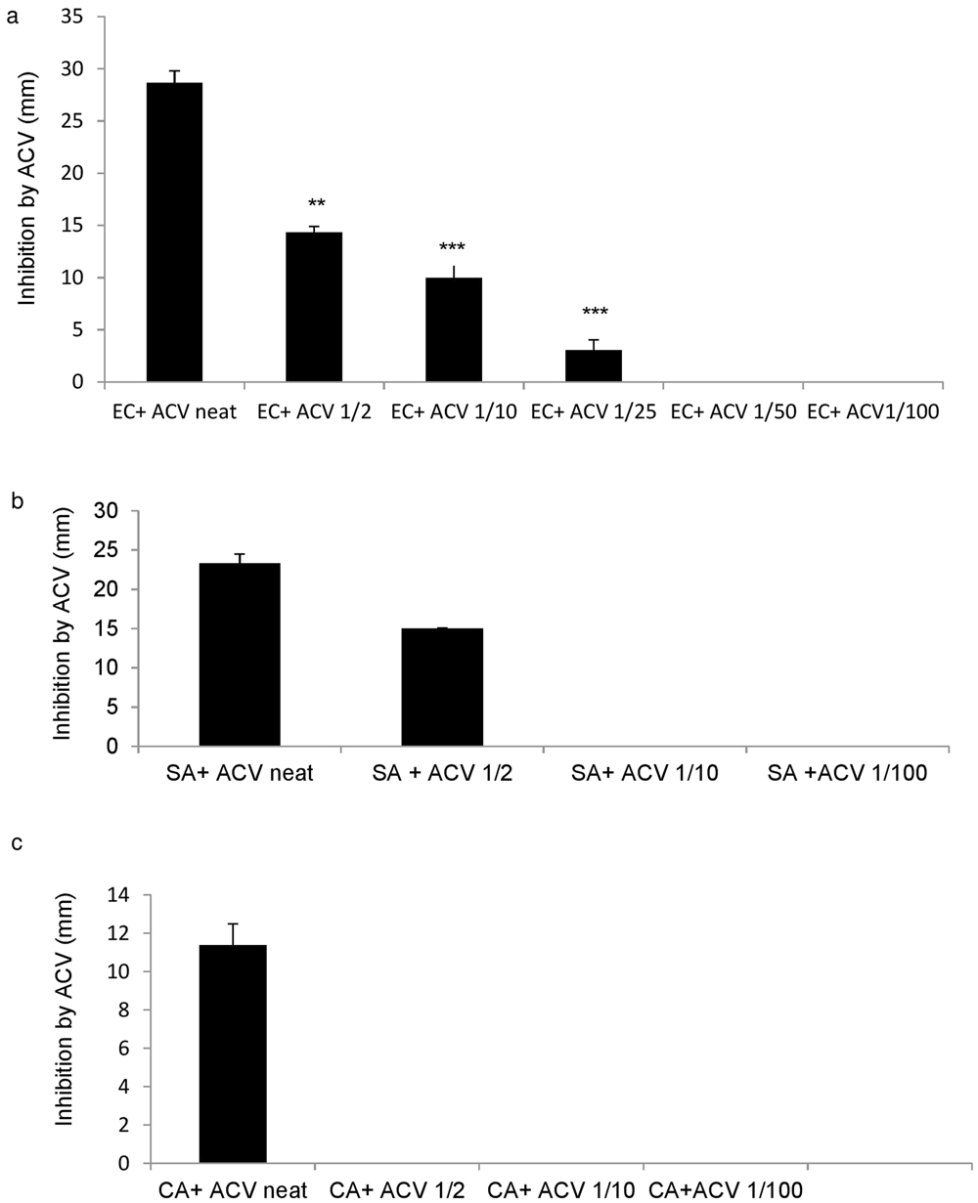
Inhibition of microbe growth by ACV after incubation for 24 h at 37 °C. (**a**) *E-coli*; (**b**) *S. aureus* (**c**) *C. albicans*. Zone of inhibition was measured in mm. These experiments represent data from three repeats. EC = *E-coli*, SA = *S*. *aureus*, CA = *C*. *albicans*, ACV = Apple cider vinegar.

In order to determine the anti-microbial activity of ACV, *E. coli, S. aureus* and *C. albicans were* directly cultured with different concentrations of ACV. Figure 1 represents the experimental results. The minimum dose required to restrict growth for *C. albicans* was neat, undiluted ACV (5% acidity), for *S. aureus* it was a 1/2 dilution (2.5% acidity) and for *E-coli*, growth was restricted at a significantly lower dilution of 1/50 (equivalent to 0.1% acidity). We also measured the equivalent zones of inhibition (in mm) for each of the microbes at varying dilutions of ACV which is depicted in the photographs of the culture plates (Fig. 2). To translate the MIC into supplementary tablet dosages required, we tested concentration ranges from 400 µg/ml in doubling microdilutions to the lowest of 3.1 µg/ml against each microbe. The MIC for ACV tablets at which no growth was visible was 62 µg/ml for *E. coli; 125* µg/ml for *S. aureus and* 250 µg/ml for *C. albicans* respectively. Both sets of results were also confirmed further by microdilution. We used the Braggs ACV for all future experiments at the minimum inhibitory dilution required for each organism.

### Downregulation of pro-inflammatory cytokine secretion by ACV in monocytes exposed to microbes

The microbes utilised in our study have been extensively studied and are known to cause inflammation through their capacity to stimulate the leucocyte pro-inflammatory cytokine cascades^19,20^. Hence, we proceeded to measure mononuclear derived TNF-α and IL-6 cytokines as indicators of inflammation which are also the markers of choice for clinical diagnosis of septic infections^21^.

**Figure 3:**
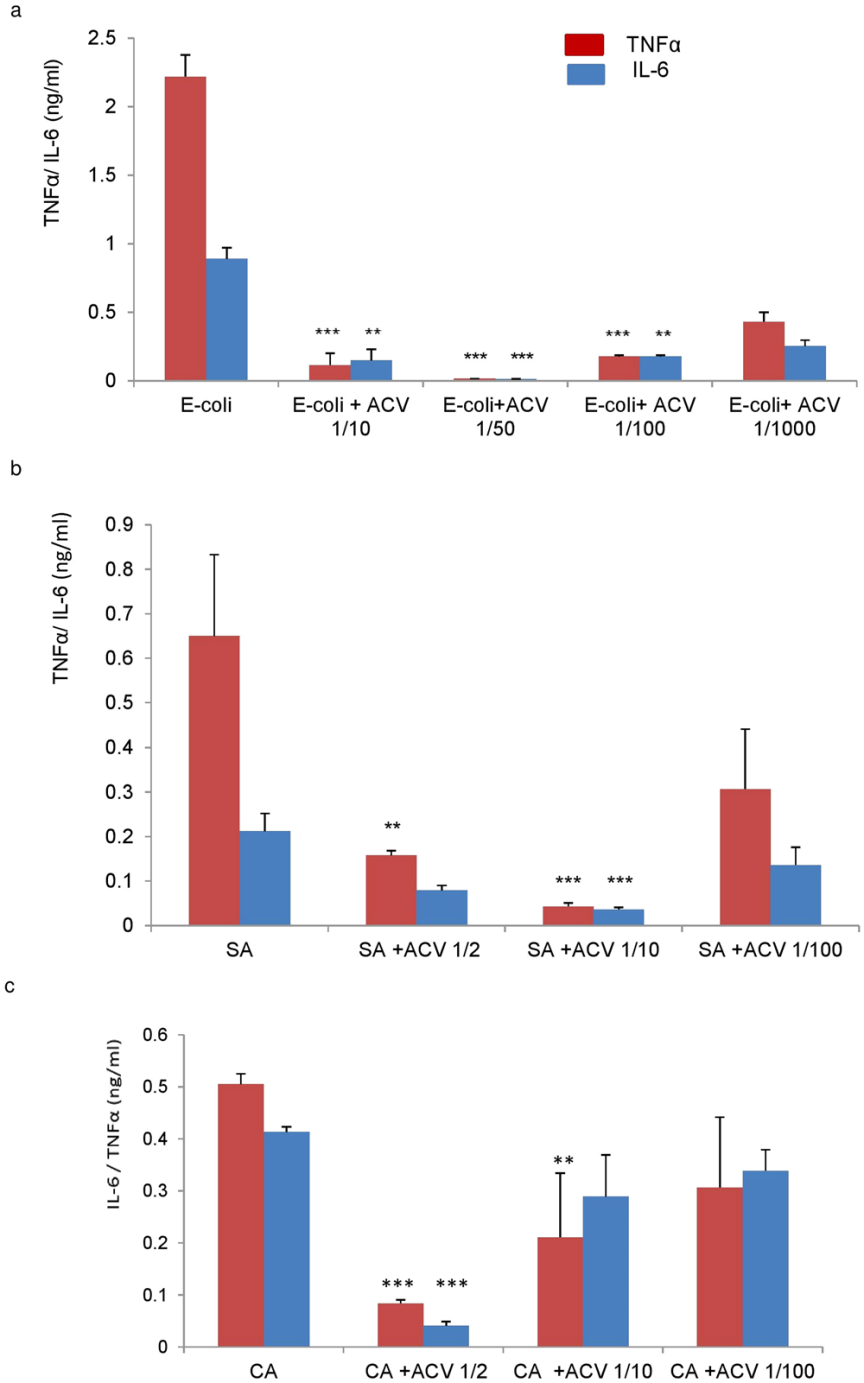
Effect of ACV on pro-inflammatory cytokine secretion from human monocytes infected with (**a**) *E-coli*; (**b**) *C. albicans* and (**c**) *S. aureus* after incubation for 24 h at 37 °C. ACV was added at dilutions of 1/10, 1/25, 1/50, 1/100 or 1/1000. EC = *E-coli*, SA = *S*. *aureus*, CA = *C. albicans*. The minimum inhibitory dilution of ACV required for significant pro-inflammatory downregulation varied with each microbe. For TNFα, a 1/50 ACV minimum inhibitory dilution was required for EC, p = 0.0008; 1/10 for SA, p = 0.01; 1/2 for CA, p = 0.0003 respectively. For IL-6, 1/50 ACV dilution was required for EC, p = 0.0008; 1/10 for SA, p = 0.03; 1/2 for CA, p = 0.008 respectively. Results represented are mean +/− SD of 3 experiments. We used student’s paired t-tests for statistical evaluation (Excel 2017) with statistical significance taken when p < 0.05. EC = *E-coli*, SA = *S. aureus*, CA = *C. albicans*, ACV = Apple cider vinegar.

Figure 3 depicts the effects of a dose dependent reduction in TNFα and IL-6 release from monocytes which have been co-cultured with ACV, together with either *C. albicans*, *E-coli* or *S. aureus* for 24 h.

**Figure 4:**
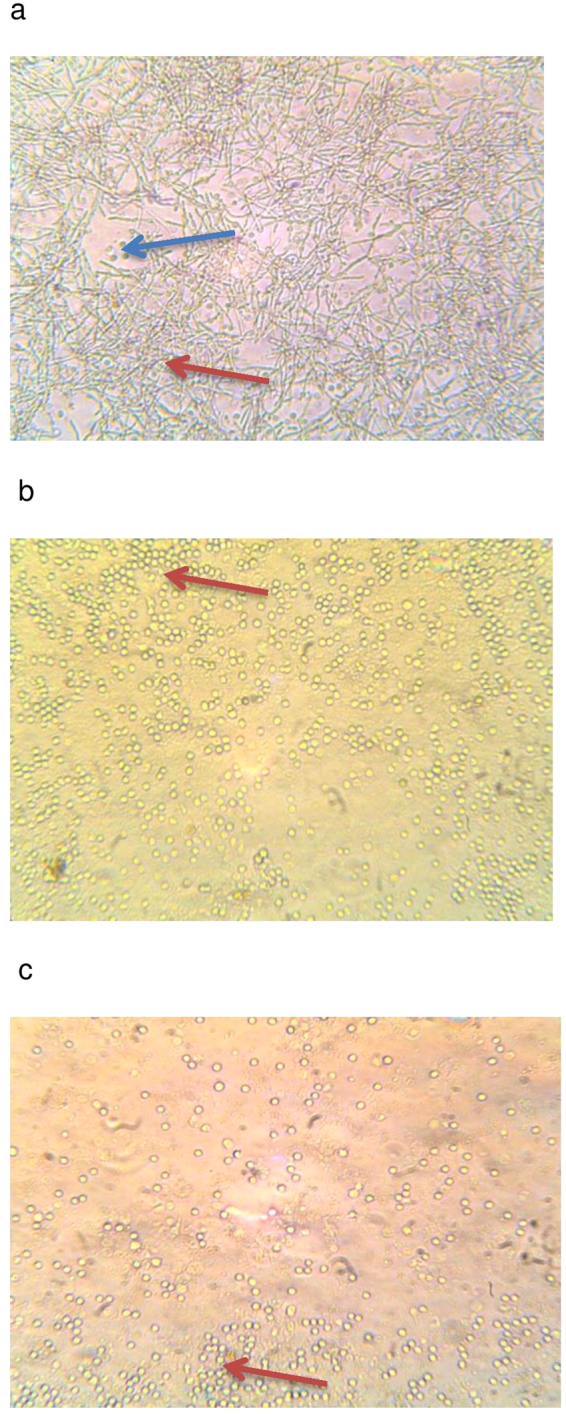
(**a**–**c**) Photos of monocytes in co-culture with microbes. Monocytes were cultured with the microbes and ACV. Trypan blue addition indicated over 95% viability. Red arrows indicate microbes and the blue arrow shows monocytes in Fig. 4a which are not visible in Fig. 4b and c since they have been covered by the microbes. Photographs were taken after 24 h incubation at 37 °C under × 100 magnification using a light microscope indicated over 90% viability with monocytes at 2, 4, 6 and 24 h of co-culture (24 h photos shown). A = *C*. *albicans*, B = *E-coli* and C = *S. aureus* respectively.

Consistent with the microbial growth inhibition data depicted in Fig. 2, the effect of ACV at the minimum inhibitory concentration of 1/50 resulted in a significant reduction in monocyte derived TNFα (p = 0.008) and IL-6 release (P = 0.001) in monocytes cultured with *E-coli*. For *S. aureus* the minimum inhibitory concentration for ACV was found to be 1/10 in terms of reduction of TNFα (p = 0.011) and IL-6 (p = 0.03). For C.albicans the minimum inhibitory dilution was lower at 1/2 dilution, for TNFα (p = 0.003) and IL-6 (p = 0.008). It was imperative to ascertain whether the monocytes were alive during inoculation with the various microbes especially after incubation for 24 h at 37 °C. We added Trypan blue directly to monocytes which had been co-cultured with microbes after 2, 6 and 24 h. Light microscopy revealed that greater than 90% of cells were alive after 24 h in all co-cultures as demonstrated in (Fig. 4a,b,c) which represents the light microscopic images of monocytes and the microbes in co-cultures.

### Upregulation of phagocytic capacity

We also investigated whether ACV could have an effect on the phagocytic function of monocytes alone and also after a 4 h exposure to microbes with or without ACV treatment. A 14.2, 13.7 and 20.4% increase in monocyte phagocytic capacity was observed after *E. coli*, *S. aureus* and *C. albicans* co-culture with ACV respectively and in comparison to the resting unstimulated monocytes. Results are expressed as the mean and SD of 3 similar experiments (Table 1). This suggests that ACV can increase phagocytic potential in monocytes which is significant as microbial phagocytosis is a key effector function of innate immunity^22^.

**Table 1:** Effect of ACV on human monocyte phagocytic capacity.

Monocyte co-culture conditions	Monocyte phagocytic capacity expressed as % change increase in side scatter. (mean ± SD)
Medium	6.1 ± 1.4
*E-coli*	9.0 ± 2.4
*S. aureus*	22.3 ± 2.2
*C albicans*	31.9 ± 2.4
*E-coli* + ACV	23.2 ± 3.7
*S. aureus* + ACV	36.0 ± 5.2
*C albicans* + ACV	52.3 ± 4.1

*In vitro* differentiated monocytes were incubated with microbes for 4 h at 37 °C. Cells cultured with microbes with or without ACV were washed and processed for detection on the Beckton Dickinson flow cytometer. An analysis of changes in regional gated profiles and % shift in side scatter was measured. Data is presented as mean ± SD of 3 similar experiments.

### Proteomic results of *E. coli*, *S*. *aureus* and *C. albicans* after exposure to ACV

The bottom-up proteomic study of ACV treated *E-coli* cultures revealed the absence in detection of key enzymes; citrate synthase, isocitrate dehydrogenase, deoxyribose–phosphate idolase, malate dehydrogenase, aminomethyltransferase and formate acetyltransferasesuccinyl-CoA ligase (Table 2). An absence in acyl carrier protein, DNA protein including DNA protection during starvation protein, integration host factor subunit alpha and ribosome associated inhibitor A was evident. Following ACV treatment *S. aureus* cultures failed to express 50 s ribosomal proteins L2, L15, L23, L24, enzymes; alcohol dehydrogenase, catalase, formate acetyltransferase, L-lactate dehydrogenase-2, ornithine aminotransferase and serine hydroxymethyl transferase (Table 3). Cell division protein ftsZ and chaperone protein Dnak were also not detected. However an important pentose phosphate pathway enzyme: 6 phosphogluconate dehydrogenase decarboxylating was displayed. Table 4 demonstrates that key enzymes required for glycolysis and candida immunogenicity were undetected after 24 h of exposure to ACV in *C. albicans*. These incorporated fructose bisphosphate aldolase, phosphogluconate dehydrogenase, pyruvate kinase and peptidyl-propyl cis-trans isomerase.

**Table 2:** List of *E-coli* proteins identified following ACV treatment. *E-coli* were cultured with 1/50 dilution of ACV or alone in broth for 24 hours at 37 °C in a shaking incubator. After which mass spectroscopy analysis was carried out.

Protein Name	Mass (Da)	Control *E-coli* culture	ACV Treated *E-coli* culture
30S ribosomal protein S1	—		*
30S ribosomal protein S11	13950		*
30S ribosomal protein S4	23512		*
30S ribosomal protein S6	15177		*
30S ribosomal protein S7	17593	*	
30S ribosomal protein S8	14175	*	
50S ribosomal protein	14923	*	*
50S ribosomal protein L1	24714		*
50S ribosomal protein L17	14413	*	*
50S ribosomal protein L2	29956	*	
50S ribosomal protein L6	18949		*
60 kDa chaperonin	57464	*	*
Acyl carrier protein	8693	*	
Aminomethyltransferase	40235	*	
Autonomous glycyl radical cofactor	—		*
Citrate synthase	48383	*	
Cytidine deaminase	31805	*	
Deoxyribose-phosphate aldolase	27958	*	
DNA protection during starvation protein	18684	*	
DNA-binding protein H-NS	15587		*
DNA-binding protein HU-alpha	9529	*	
DNA-binding protein HU-beta	9220	*	
Elongation factor Tu 1	43427	*	*
Enolase	45683	*	*
Formate acetyltransferase 1	85588	*	
Glutamate/aspartate periplasmic-binding protein	33513	*	
Glyceraldehyde-3-phosphate dehydrogenase	35681	*	*
Integration host factor subunit alpha	11347	*	
Isocitrate dehydrogenase [NADP]	46070	*	
Major outer membrane lipoprotein Lpp	8375		*
Malate dehydrogenase	32488	*	
Outer membrane protein A	37292	*	*
Ribosome-associated inhibitor A	12777	*	
Succinate dehydrogenase flavoprotein subunit	65008	*	*
Succinyl-CoA ligase [ADP-forming] subunit beta	42244	*	
Transaldolase B	35368		*
Uridine phosphorylase	27313	*	*

The “*” indicates the presence of protein whilst the blank region denotes no detection of that particular protein.

**Table 3:** List of *S. aureus* proteins identified following ACV treatment.

Protein name	Mass (Da)	Control *S. aureus* culture	ACV- Treated *S. aureus* culture
30S ribosomal protein S1	43250	*	*
30S ribosomal protein S12	15334	*	
30S ribosomal protein S2	29133	*	
30S ribosomal protein S3	24085	*	*
30S ribosomal protein S4	22999	*	
30S ribosomal protein S5	17732	*	
30S ribosomal protein S6	11588	*	
30S ribosomal protein S7	17783	*	*
30S ribosomal protein S8	14822	*	
50S ribosomal protein L1	24693	*	
50S ribosomal protein L13	16323	*	*
50S ribosomal protein L14	13241	*	*
50S ribosomal protein L15	15587	*	
50S ribosomal protein L2	30194	*	
50S ribosomal protein L21	11326	*	*
50S ribosomal protein L23	10599	*	
50S ribosomal protein L24	11529	*	
50S ribosomal protein L25	23773	*	*
50S ribosomal protein L29	8085		*
50S ribosomal protein L4	22451	*	*
50S ribosomal protein L6	19774		*
6-phosphogluconate dehydrogenase, decarboxylating	51941		*
Alcohol dehydrogenase	36424	*	
Arginine deiminase	47113	*	*
Bacterial non-heme ferritin	23773	*	*
Catalase	58457	*	
Cell division protein FtsZ	41012	*	
Chaperone protein DnaK	66338	*	
DNA-binding protein HU	9620	*	*
Elongation factor Tu	43134	*	*
Enolase	47145	*	*
ESAT-6 secretion system extracellular protein A	11029	*	
Formate acetyltransferase	85264	*	
Fructose-bisphosphate aldolase class 1	32907		*
Isocitrate dehydrogenase [NADP]	46451		*
L-lactate dehydrogenase 2	34468	*	
Ornithine aminotransferase 2	43675	*	
Ornithine carbamoyltransferase, catabolic	37853	*	*
Probable malate:quinone oxidoreductase	56135	*	*
Putative universal stress protein SA1532	18521	*	*
Pyruvate dehydrogenase E1 component subunit beta	35194	*	*
Serine hydroxymethyltransferase	45384	*	

*S. aureus* were cultured with 1/10 dilution of ACV or alone in broth for 24 hours at 37 °C in a shaking incubator. After which mass spectroscopy analysis was carried out. The “*” indicates the presence of protein whilst the blank region denotes no detection of that particular protein.

**Table 4:** List of *C. albicans* proteins identified following ACV treatment.

Protein name	Mass (Da)	Control *C.albicans* culture	ACV- Treated *C.albicans* culture
40S ribosomal protein S1	29083	*	*
6-phosphogluconate dehydrogenase	71270	*	
Alcohol dehydrogenase 1	37255	*	*
Elongation factor 1-alpha 1	50436	*	*
Elongation factor 2	93865	*	*
Enolase 1	47202	*	*
Fructose-bisphosphate aldolase	39362	*	
Glucose-6-phosphate isomerase	61148		*
Glyceraldehyde-3-phosphate dehydrogenase	35925	*	*
Heat shock protein SSA1	70452	*	*
Mitochondrial outer membrane protein porin	29748		*
Peptidyl-prolyl cis-trans isomerase	17678	*	
Phosphoglycerate kinase	45266	*	*
Phosphoglycerate mutase	27437	*	*
Plasma membrane ATPase 1	98083	*	*
Pyruvate decarboxylase	62744	*	*
Pyruvate kinase	55752	*	
Small heat shock protein 21	21482	*	*
Triosephosphate isomerase	26880	*	*
White colony protein WHS11	6991	*	

*C*. *albicans* were cultured with 1/2 dilution of ACV or alone in broth for 24 hours at 37 °C in a shaking incubator. After which mass spectroscopy analysis was carried out. The “*” indicates the presence of protein whilst the blank region denotes no detection of that particular protein.

## Discussion

ACV has multiple antimicrobial properties on different microbial species, affecting microbe growth, suppressing mononuclear cytokine and phagocytic responses. The tandem mass spectroscopy results are in cohesion with these observations. The microbes underwent significant impairment following ACV addition which damaged cell integrity, structural and metabolic proteins as well as nuclear material. Indeed the enzymes citrate synthase, isocitrate dehydrogenase, malate dehydrogenase, aminomethyltransferase and formate acetyltransferasesuccinyl-CoA ligase are crucial for *E-coli* growth, gene regulation and central, intracellular carbon metabolism. An absence of these enzymes would affect glycolytic, tricarboxylic acid cycle, pentose phosphate, glycoxlate shunt and oxidative phosphorylation pathways in *E. coli*^23^. Furthermore, the absence of DNA binding protein from starved cells, is significant as it protects *E-coli* and functions to control gene regulation during cell starvation^24^. With respect to other proteins, we observed the presence of ribosomal proteins (L1, L6 and 30 s ribosomal proteins (S1, S4, S6, S7, S8, S11) in ACV treated *E. coli* compared to control *E. coli*. These are mostly RNA binding proteins hence their presence could have been due to partial disintegration of 50 s and 30 s ribosomal breakdown whereas these subunits might remain intact in untreated *E. coli* cultures. The absence of ribosome associated inhibitor A could interrupt *E. coli* growth cycles as it serves to minimise translational errors^25^. Collectively these results support the cytotoxic effects of ACV we observed on *E-coli*. ACV treated *S. aureus* cultures did not express the chaperone protein Dnak and the cell division protein ftsz. This is significant as previous studies have shown that a non-functional DnaK system can cause a reduced tolerance to heat, oxidative, antibiotic stresses and lowered carotenoid production^26^. There was also an absence of gateway enzymes involved in multiple pathways such as ornithine aminotransferase^27^. 6 phosphogluconate dehydrogenase decarboxylating was expressed which is not surprising as it plays a critical role in protecting cells from oxidative stress^28^. The effect of ACV on *C.albican* protein moieties was less dramatic, nevertheless we did detect the absence of key enzymes which are fundamental in maintaining cell integrity and biosynthetic pathways^29^.

There could be a strong possibility that ACV acts like other anti-pathogenic compounds in diverting monocyte responses through toll receptor signalling pathways. There is evidence that *E-coli in particular* can induce a typical M1 monocyte profile through mechanisms involving NF-kB activation. This results in upregulation of inflammatory cytokines TNF-α and PI3 kinase stimulation^30^. The *in vitro* M1 monocyte phenotype is also prominent in severe sepsis. This was shown in a study using baboons where a substantial mortality rate correlated with high serum levels of TNFα and IL-6 following induced sepsis infection^31^. Unchecked high levels of circulating M1 cytokines can rapidly lead to cardiac arrest and death hence any factor capable of lowering pro-inflammatory cytokine concentrations is essential in therapy^32^. It has been reported that ACV consists of acetic acid, flavonoids such as gallic acid, tyrosol catechin, epicatechin, benzoic acid, vaninilin, caftaric acid, coutaric acid, caffeic acid, acid and ferrulic acid These constituents have been reported to affect immune defence and oxidative responses^18,33^.

Furthermore, the mechanism of ACV activity could be attributed in part to the apple polyphenol content. Yang *et al*. (2010) reported on the cellular protective effects of apple polyphenols on induced liver damage whereby histopathological tissue destruction was limited and liver activity maintained in mice that received the polyphenols^34^. The mechanisms involved were free radical scavenger action, lipid peroxidation modulation and the antioxidant upregulation capacity of ACV. Interestingly, a study by Denis *et al*. demonstrated the anti-inflammatory potential of apple phenols on gastrointestinal cell inflammation which involved downregulation of TNF and IL-6 cytokines^35^. Another means of action could involve the acetic acid component of ACV which is able to reduce the cell hydrogen potential hence could potentially facilitate diffusion across the plasma membrane of microbes. Furthermore, there is evidence that organic acids can alter immune responses by binding to GPR3, a G protein coupled receptor which is mostly expressed on inflammatory leukocytes^36^. Also, an investigation reported on upregulated blood and plasma antioxidant enzyme release after apple consumption which would encourage immune protection^16^.

The positive benefits of dietary ACV supplementation have been highlighted *in vivo*. ACV decreased the serum lipid profile in mice fed a high cholesterol diet over 28 days. Intragastric ACV addition induced a protective effect against erythrocyte, kidney and liver oxidative injury as well as lowering cholesterol levels^18^. ACV also decreased blood triglyceride and very low density lipoprotein levels in rats which had induced cholesterol induced hepatic steatosis^33^. In an infection induced model of denture stomatitis, ACV addition resulted in anti-fungal activity against Candida Spp which was comparable to nystatin in terms of reducing microbial adherence and destruction^37^.

Severe infections, autoimmunity or transplantation can inevitably lead to ineffective immunity in patients. An analysis of macrophages from Crohn’s disease patients revealed that they had defective responses to *E-coli* due to Crohn’s related systemic immunosuppression^38^. A recent report showed that co-administration of ACV with L-*casei* boosted systemic and mucosal immune responses, antioxidant enzyme and growth genes in fish^39^. Equally, perhaps additive dietary supplementation with ACV could be of benefit in acute infections, autoimmune induced immune dysregulation or antibiotic redundancy in humans. Future studies would establish whether ACV could be used as a potential therapeutic, *In vivo* models of infection could be induced by infusing microbes systemically into mice followed by treatment with or without intraperitoneal ACV. Intragastric ACV has been fed to animals used as models of obesity and infection in the past^18,37,39^. ACV efficacy could be evaluated by measuring microbial burden, serum cytokine levels, leukocyte counts and tissue pathology. Side effects could include acid reflux, nausea or delayed digestion as ACV has a pH of 4.2. However, the acidity could be neutralised by the addition of sodium bicarbonate to preparations. The results of this study could have clinical implications as ACV could be used as an additive component of an antimicrobial therapeutic regimen especially in immunocompromised patients presenting with infections of the aforementioned microbes.

We conclude that ACV can have multiple antimicrobial effects directly on *E-coli*, *S. aureus* and *C. albicans*. ACV addition can also decrease induced inflammatory cytokine release during mononuclear leukocyte infection and increases monocyte phagocytic capacity. Mechanisms include alteration of the microbial protein physiology destroying structural pathogenic proteins and metabolic enzymes. Collectively our results highlight the potent antimicrobial and therefore beneficial actions of ACV. This preliminary study encourages further work on dietary ACV supplementation investigating its antimicrobial role and the constituents which could be responsible for this activity.

## Materials and Methods

### Chemical reagents, microorganisms, media and culture conditions

A selection of microbial specimens which represented a typical gram positive, a gram negative and a yeast species were chosen for initial investigation. Microbial strains: *E-coli* strain NCTC 10418 and *S. aureus* strains NCTC 6571 were purchased from Health Protection Agency (Colindale, U.K.). *C. albicans* strain 90828 was purchased from the American Type Culture Collection (LGC Promochem).

#### Reagents

Dulbecco’s modified media, dimethyl-ethyl-sulphonyl-oxide, HANKS balanced salt solution, histopaque, ethanol, phosphate buffered saline, paraformaldehyde, acetone, dithiothreitol, iodoacetamide, trypsin from porcine pancreas of proteomics grade, formic acid, acetonitrile, HPLC-grade water, methanol and Whatman Mini-UniPrep syringeless filter devices (pore size 0.45 µm) were purchased from Sigma Aldrich (Poole, U.K.). TNF-alpha, interleukin-6 (IL-6) enzyme linked immunosorbent assays (ELISA’s) were purchased from Research and Development Systems (Abingdon, U.K.). Mueller hinton agar was purchased from Oxoid, UK. Braag’s Apple Cider Vinegar and apple cider vinegar tablets (500 mg, Troo healthcare) were purchased from commercial sources.

### Inoculum preparation and measurement of anti-microbial activity of ACV

Cultures of *E. coli* and *S. aureus* were grown in nutrient media whereas *C. albican*s was grown in Sabourand media. All cultures were cultivated in a shaking incubator at 37 °C for 24 h overnight prior to use. Mueller hinton agar (MHA) was prepared by dissolving 38 g in 1 litre of distilled water, boiling the mixture for 1 min, after cooling and autoclaving, the media was poured into petri dishes. The plates were left to dry and subsequently stored at 37 °C. All microbial cultures were adjusted to 0.5 McFarland’s standard 1.5 × 10^8^ CFU/ml and 4 × 10^6^ CFU/ml of each organism used in experiments. Each microbe was swabbed evenly onto plates containing MHA. For sample addition, 100 µL of ACV at varying concentrations was added to the wells which were punched into the agar. The plates were then incubated at 37 °C for 24 h. Zones of inhibition surrounding samples were identified, photographed and measured in mm^40^. Experiments were repeated at least 5 times.

### Ethical Approval and Informed Consent

All experimental protocols were approved by the Middlesex University Natural Sciences Ethics Committee number 2323. Further the methods were carried out in accordance to the relevant guidelines and regulations. Informed consent was received when applicable.

### Human mononuclear cell isolation procedure from whole peripheral blood

Human leucocyte rich cones and serum were obtained from volunteer donors collected from NHS Cord blood and transplant bank at Colindale, London and treated as described previously^41^. Briefly the cones were washed with phosphate buffer saline to harvest the leucocyte rich cells. These were then spun on histopaque density gradient at 1200 RPM for 20 min. Monocytes were purified using the CD14 positive mononuclear portion which was isolated according to manufacturer’s instruction provided with the pan monocyte isolation kit. Cells washed with HANKS balanced solution, counted and cultured into 24 well plates at 4 × 10^5^ cells per mL. Cells were allowed to adhere for an hour at after which non-adherent cells were washed away and full media replenished with Dulbecco’s media containing 10% human serum. Monocytes were determined using light microscopy and flow cytometry phenotypic analysis of differentiation markers as described previously using CD14^41^. Freshly isolated monocytes were cultured with varying concentrations of ACV and either *C. albicans*, *E*. *coli* or *S*. *aureus* at counts of 4 × 10^6^ CFU/ml respectively for 24 h at 37 °C and 5% CO_2_ after which supernatants were collected and analysed for TNF-α or IL-6 secretion using ELISA kits following manufacturer’s protocols.

### Monocyte phagocytic capacity measurement by flow cytometry

Isolated human mononuclear cells were cultured at 4 × 10^5^/mL in 24 well plates over a period of two days after which they were incubated with microbes (4 × 10^6^ CFU/ml) for 4 h at 37 °C and 5 CO_2_. Cells were then scraped replenished in ice cold PBS containing 1 mM EDTA, washed and removed from plates. The resultant pellets were fixed in 400 µL of 4% paraformaldehyde and analysed using a FACS Calibur flow cytometer (Beckton Dickinson Immunocytometry Systems, UK and *Cell Quest* software).

### Preparation of microbial tryptic digests for mass spectroscopy analysis

An aliquot of microbial suspension was collected and treated over 24 h with 1/100 ACV, washed with PBS, resuspended in PBS and then treated with 1 mL of ice-cold acetone. The bacterial cells were harvested after centrifugation 13,000 g for 5 min. The pellet was dried and then reconstituted in 50 mM ammonium bicarbonate. Cells were lysed using a Soniprep 150 Plus (MSE, U.K.) for 10 s with the amplitude set at 13. Subsequently proteins were denatured and reduced with 3 μL of 100 mM dithiothreitol in 50 mM ammonium bicarbonate at 95 °C for 5 min followed by alkylation with 6 μL of 100 mM iodoacetamide in the dark at room temperature for 20 min. Proteins were then digested with 2 μL of trypsin (0.1 μg/μL dissolved in 50 mM ammonium bicarbonate) at 37 °C for 3 h. A further 2 μL of trypsin was added to the sample and the mixture was incubated at 37 °C for an additional 2 h. The samples were diluted in 150 µL 50 mM ammonium bicarbonate and passed through Mini-Uni Prep filter devices.

### Liquid Chromatography-Electrospray Ionisation Tandem Mass Spectrometry

The tryptic peptides were analysed using a Shimadzu Prominence HPLC system hyphenated to an electrospray ionisation hybrid ion-trap time-of-flight (IT-TOF) mass spectrometer (Shimadzu, U.K.) operated in tandem mass spectrometry mode. Peptides were separated using an Ascentis Express 150 × 2.1 mm, 2.7 µm C18 column (Sigma-Aldrich, Poole, U.K.) using a flow rate of 0.21 mL/min. The column oven temperature was set to 40 °C. Data was acquired and processed using LabSolutions®software (version 3.50.348, Shimadzu, UK). A linear gradient elution profile composed of ‘A’−0.1% formic acid in water and ‘B’- 0.1% formic acid in acetonitrile was used. The gradient profile was 0–40% B, 70 min; 40–90% B, 1 min; maintained at 90% B, for 3 min; 90–0% B, 1 min; and 15 min re-equilibration at 0% B. An injection volume of 40 µL was used. Samples were kept in the auto sampler set to 4 °C. Mass spectrometry analysis was performed in MS/MS mode using positive ions electrospray. The precursors’ acquisition range was set to 400–1,800 *m/z* while the fragments acquisition range was set to 200–1,500 *m/z*. For both precursors and fragments the ion accumulation time was set to 30 msec. The other instrument conditions were set as follows: detector voltage 1.6 kV, CID energy 70%, nebulising gas flow 1.5 L/min, CDL temperature 200 °C, heat block temperature 200 °C, interface voltage 4.5 V, detector voltage 2 kV. The data acquisition was performed in a 37.5 min interval.

### LC-MS/MS Data Processing

MS/MS data were extracted from the resulting instrument files using Mascot Distiller software (version 2.5.1.0, Matrix Science, London, UK). For precursors peak picking the following parameters were used: correlation threshold – 0.7, minimum signal to noise ratio – 5, minimum peak *m/z* – 50, maximum peak *m/z* – 100,000, minimum peak width – 0.02 Da, expected peak width – 0.2 Da, maximum peak width – 2 Da. The MS/MS ion list was searched using Mascot search engine against all entries in Swiss-Prot database (2016_2). For database search the following parameters were used: two missed cleavages, carbamidomethylation of cysteine (as fixed modification) and oxidation of methionine (as variable modification). The tolerance for precursor peptides was set to 10 ppm and for fragments to 0.3 Da. Peptide charges used for peak picking was +2, +3 and +4.

### Statistical analysis

All experimental results are expressed as the mean ± standard deviation (SD). Statistical analyses was carried out using one way ANOVA or students t-test, outcomes were considered significant where p < 0.05 (when comparing apple cider vinegar treated microbes to the untreated groups in all experiments). All experiments were repeated at least 3–5 times. Analysis was carried out using Excel software version 2016.

### Data availability

The datasets generated and analyzed during the current study reside with the corresponding author and can be made available upon request.
